# Network-based protein-protein interaction prediction method maps perturbations of cancer interactome

**DOI:** 10.1371/journal.pgen.1009869

**Published:** 2021-11-02

**Authors:** Jiajun Qiu, Kui Chen, Chunlong Zhong, Sihao Zhu, Xiao Ma

**Affiliations:** 1 Shanghai Children’s Hospital, Shanghai Institute of Medical Genetics, Shanghai Jiao Tong University, Shanghai, China; 2 NHC Key Laboratory of Medical Embryogenesis and Developmental Molecular Biology & Shanghai Key Laboratory of Embryo and Reproduction Engineering, Shanghai, China; 3 Department of Neurosurgery, Shanghai East Hospital, Tongji University School of Medicine, Shanghai, China; 4 Department of Biomedical Engineering, College of Engineering, Peking University, Peking, China; 5 Research Group Cell Signalling, Department of Psychiatry, Ludwig Maximilian University of Munich, Munich, Germany; Peking University, CHINA

## Abstract

The perturbations of protein-protein interactions (PPIs) were found to be the main cause of cancer. Previous PPI prediction methods which were trained with non-disease general PPI data were not compatible to map the PPI network in cancer. Therefore, we established a novel cancer specific PPI prediction method dubbed NECARE, which was based on relational graph convolutional network (R-GCN) with knowledge-based features. It achieved the best performance with a Matthews correlation coefficient (MCC) = 0.84±0.03 and an F1 = 91±2% compared with other methods. With NECARE, we mapped the cancer interactome atlas and revealed that the perturbations of PPIs were enriched on 1362 genes, which were named cancer hub genes. Those genes were found to over-represent with mutations occurring at protein-macromolecules binding interfaces. Furthermore, over 56% of cancer treatment-related genes belonged to hub genes and they were significantly related to the prognosis of 32 types of cancers. Finally, by coimmunoprecipitation, we confirmed that the NECARE prediction method was highly reliable with a 90% accuracy. Overall, we provided the novel network-based cancer protein-protein interaction prediction method and mapped the perturbation of cancer interactome. NECARE is available at: https://github.com/JiajunQiu/NECARE.

## Author summary

Protein-protein interaction (PPI) network is the biological foundation for the normal function of cells, while the perturbation of this network can result in the pathological state, such as cancer. Notably, the perturbation of PPI network in cancer not only involves in the destruction of old PPI, but also the reconstruction of new PPIs. However, due to the limit of tools, instead of the real physical interaction between proteins, previous cancer network researches only focus on the co-expression relationships. Now, with the development of computational biology, we established a novel cancer specific physical PPI prediction method dubbed NECARE, which was based on relational graph convolutional network (R-GCN) with knowledge-based features. It can infer the PPI in cancer from a general network. And we reveal the cancer PPI interactome by doing high-throughput analysis with NECARE. Also, many cancer hub genes were identified during the analysis, which were enriched for cancer network perturbations. Future studies can benefit from both our method itself and the results of our analysis.

## Introduction

**Fig 1:**
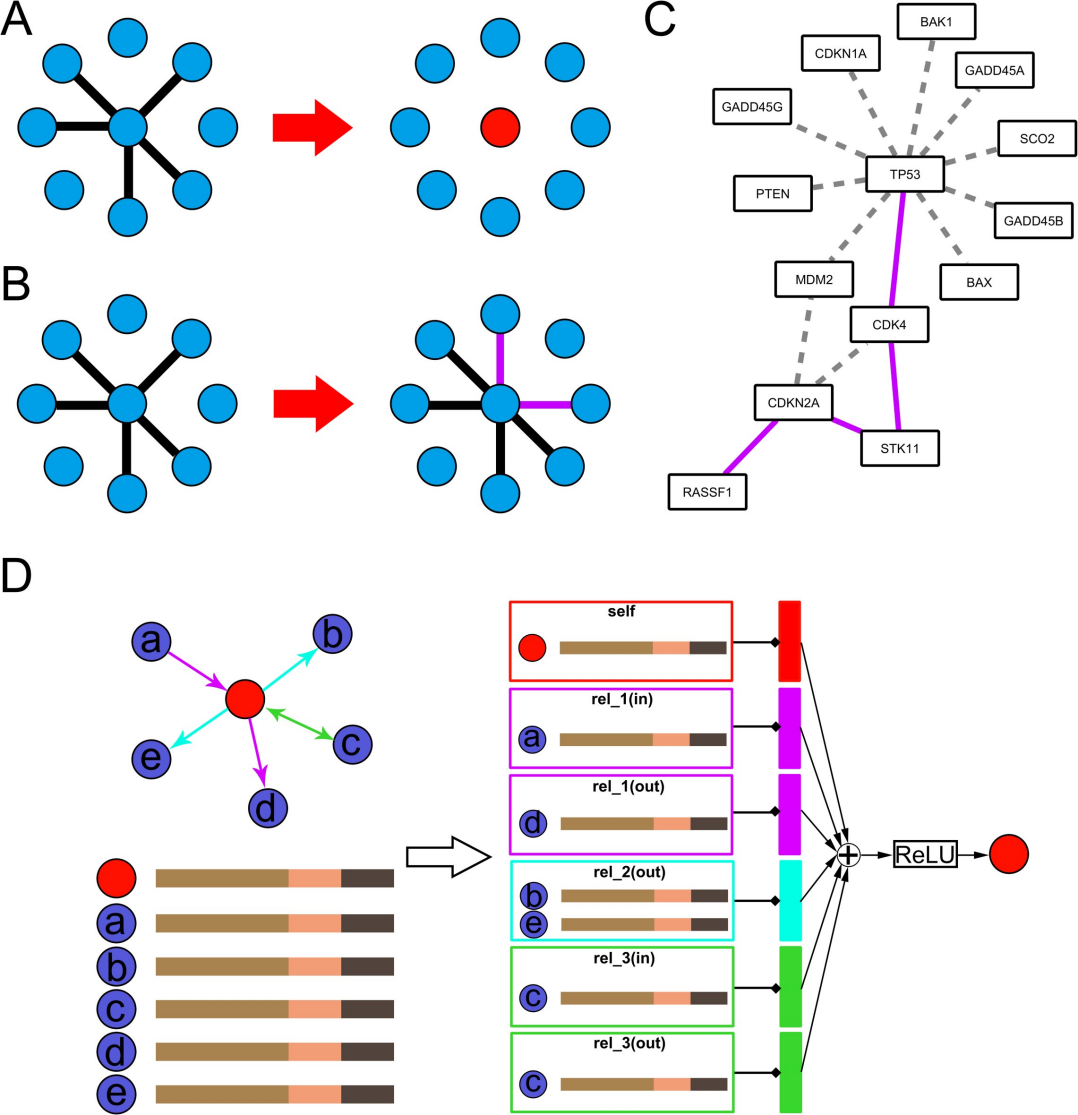
Illustration of the perturbation of the protein relationship network and NECARE algorithm. **Panel A-C** introduce the concept of protein network perturbation. (A) Each node represents a protein. Mutations such as nonsense mutations could cause the node to be totally inactive or absent (red) and lose all the edges connected to this node (gray dashed edges). (B) Each node represents a protein. Mutations such as missense mutations could cause the gain or loss of specific edges (purple edges mean the new gained edges due to the mutations; gray dashed edge means lost interaction), while the center node is not totally inactive. (C) This is an example of the perturbation of the protein relationship network in cancer. The example is based on the KEGG database (6). Gray dashed edges are the interactions that are lost in cancer, and purple edges are the new interactions in which genes are involved in cancer. **Panel D** is a simple example to show how we represent the gene (red node) by NECARE with R-GCN. Nodes a-e and the red node represent different genes, and the red node is set as the target gene. Nodes a-e are all in contact with the red node, and different colored edges represent different types of interactions. First, each node is represented by a feature vector that contains three parts: (tan: OPA2Vec; salmon: TCGA-based expression feature; and taupe: TCGA-based mutation feature). Then, to represent the red node, the feature vectors are gathered and transformed for each relation type individually (for both in- and out-edges; also, a self-loop is included). The resulted representation (vertical rectangles with different colours for different relationship types) is summed up and passed to an activation function (ReLU).

Cells are biological systems that employ a large number of genes and signaling pathways to coordinate multiple functions [1]. Therefore, instead of acting in isolation, genes interact with each other and work as part of complex networks [2]. The completeness of these networks is the foundation of the normal biological systems, while perturbation of them can result in the pathological state. Recent studies have already found network perturbation is the cause of cancers, rather than the dysregulation of single proteins [2]. Protein network in cancer is perturbed by many factors, one of which could be mutations. Disease-causing mutations can not only produce a mutated gene and thus a mutated protein, but also disturb the interactions between the mutated protein and its normal molecular partners [3]. Additionally, distinct mutations will cause different molecular defects in proteins, and they may lead to distinct perturbations of protein networks, giving rise to distinct phenotypic outcomes [4]. Nonsense mutations that grossly destabilize a protein structure can be modeled as removing a protein node from the network (Fig 1A). Alternatively, missense mutations may give rise to partially functional protein products with specific changes in distinct biophysical or biochemical interactions (Fig 1B) [4]. Furthermore, studies have already found that missense mutations in cancer are more likely to occur on the interaction interface of proteins. Thus, network perturbation, instead of single protein dysregulation, has been found to be the reason for human diseases, especially cancers [5]. For example, in cancer, TP53, a well-known tumor suppressor protein (Fig 1C), loses many interactions with other important proteins, such as PTEN and MDM2 [6]. However, new proteins, such as CDK4, have been discovered to interact with TP53. In the normal network, the cross-talk line from TP53 to CDKN2A is TP53-MDM2-CDKN2A, but in cancer, the cross-talk line is TP53-CDK4-STK11-CDKN2A [7]. Therefore, in cancer, mutations lead to reconstruction of the protein network rather than the simple destruction, making the protein network in cancer tissues very different from that in normal tissues.

There have been some studies about cancer network perturbations [2,8–11]. For example, James West et al. tried to identify genes with network perturbations by calculating the network entropy [10]. Maxim Grechkin et al. also identified perturbed genes through inferred gene regulators and their expression [2]. As these studies were based on only the coexpression of genes, their network was more likely to reflect the relationships (expression and repression) between transcriptional factors and their targets. However, these studies failed to consider physical relationships such as protein-protein interactions (PPIs), which are significantly different from coexpression networks based on topological comparisons [12].

As to PPIs, there has already existed different kinds of PPIs prediction methods, but they are only for non-disease situation. Generally, they fall into three categories: 1) **Structure-based methods**, which are based on the 3D structure of proteins and limited to proteins with PDB structures [13–16]. Structure-based methods are better at predicting physical interactions. 2) **Sequence-based prediction methods,** which attempt to predict interactions by the sequences of two candidate proteins [17–20]. 3) **Network-based methods** that predict interactions based on the known network. Unlike other methods which only consider two candidate proteins, network-based methods also consider their known neighbors [21–27].

In our study, we established a novel cancer PPI prediction method, dubbed NECARE (**ne**twork-based **ca**ncer PPI p**re**diction), to investigate the whole cancer PPI map. Here we applied a relational graph convolutional network (R-GCN) with knowledge-based features. One crucial novelty of this work is that, unlike previous network-based node relationship prediction algorithms, NECARE considers the type and direction of gene links at the input space, so that NECARE is able to infer the possible PPIs through gene relationships such as activation, expression, and phosphorylation. And NECARE was found to outperform the other algorithms (both network- and sequence-based algorithms) in predicting cancer PPIs. Thus, our tool can help other researchers to determine the possible upstream and downstream molecular partners of their target proteins in cancer.

Furthermore, we mapped the cancer interactome and analyzed the perturbations of PPIs in cancer with NECARE. We found that the PPI perturbations were enriched in some specific genes that were defined as cancer hub genes in our study. These hub genes were significantly related to the prognosis of 32 types of cancers. Many of these hub genes have already been well studied in previous cancer studies or served as drug targets. These findings indicated that our results can potentially provide the targets for future cancer studies. Finally, we selected 20 pairs of PPIs and verified the interaction of 18 pairs by coimmunoprecipitation, which demonstrated that NECARE prediction method was highly reliable with a 90% accuracy.

## Results

### Establish network-based cancer protein-protein network prediction method (NECARE)

**Fig 2:**
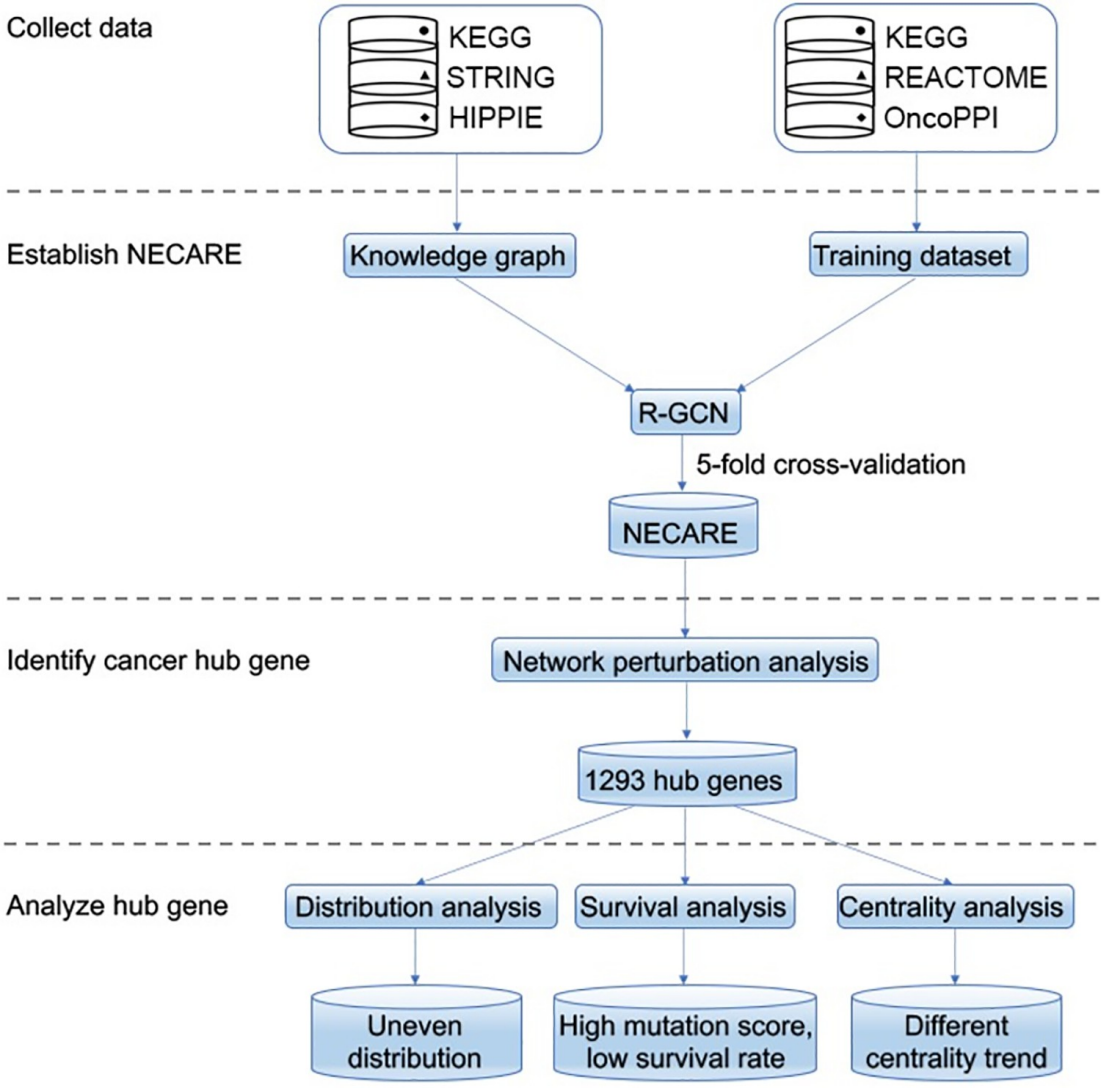
Workflow of this study. It describes the dataset we used and the whole pipeline of the research: from data collection and NECARE model training to the following network analysis with NECARE.

The PPI network in cancer is different from that in normal (non-cancer) situations. To reveal PPI network perturbation in cancer, we designed the novel network-based cancer-specific PPI prediction method: NECARE (Fig 2). The R-GCN based NECARE is different from previous network-based algorithms (such as GCN): it accounts for the type and direction of edges at the input space (Fig 1D, details seen in Materials and Methods). Basically, instead of only looking at the particular nodes (proteins/genes), NECARE also obtained the relationship information of its neighboring nodes. For example, if both protein A and protein B can regulate the expression of protein C, then it is highly possible that there is a PPI existing between protein A and protein B. Our results confirmed that, at cross-validation, R-GCN based NECARE had a significant higher performance (MCC) than that using GCN which does not consider the information of the type and direction of edges at the input space (S1 Fig). Besides, we also confirmed that using gene network (such as expression regulation and methylation etc.) at the input space was better than simply using PPI network (S1 Fig).

In our study, we tested two kinds of features for the neural network: 1) ontology-based features (OPA2Vec) and 2) TCGA-based expression and mutation profiles. Their performance was compared in the cross-training set (S2 Fig). The combination of OPA2Vec and TCGA worked better than each of them alone, reaching an MCC = 0.85 (S1 Fig). Thus, the combination of OPA2Vec- and TCGA-based (expression and mutation) profiles was selected as the features for NECARE.

**Fig 3:**
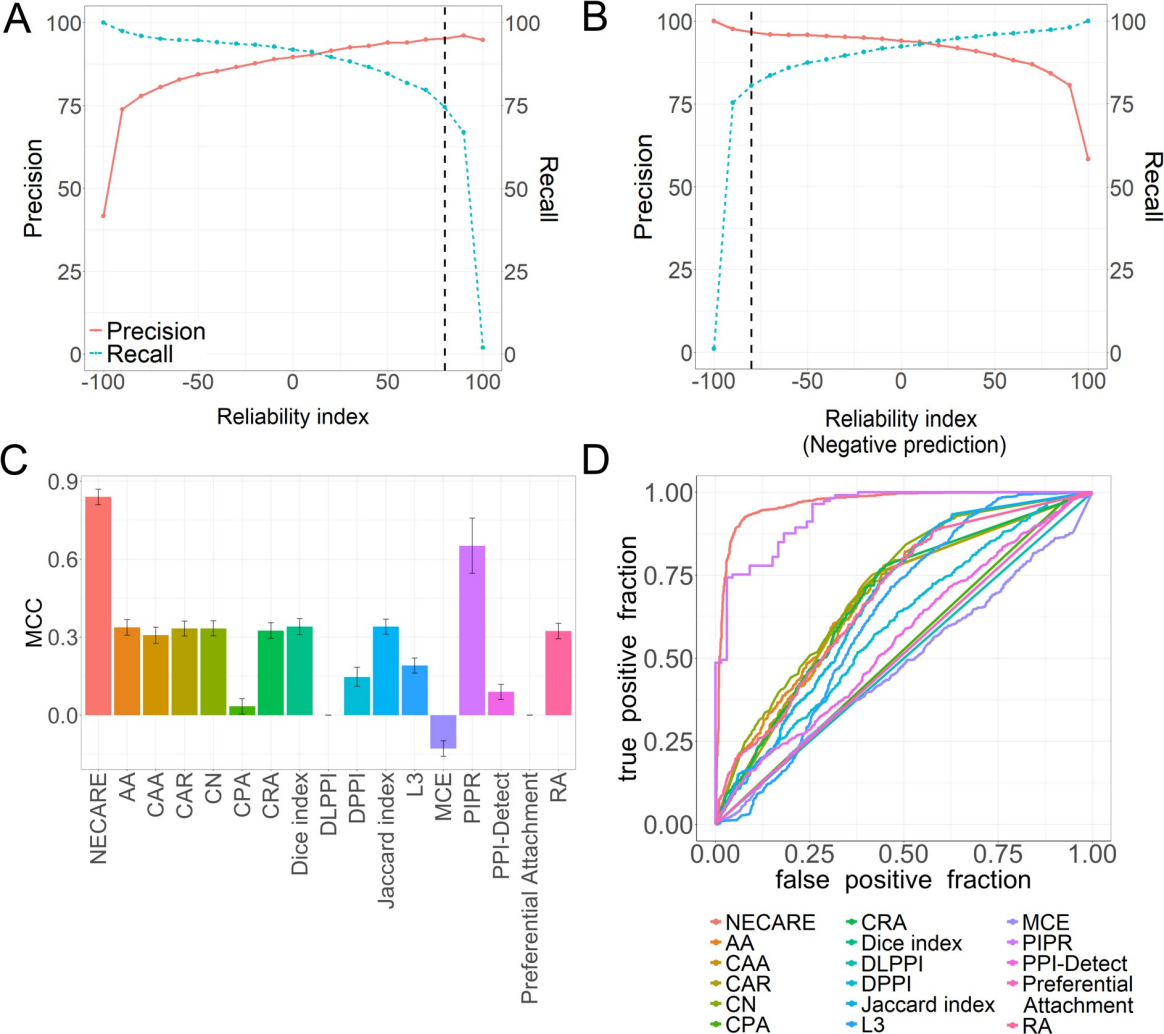
Network-based cancer gene relationship (NECARE) prediction. (A) All machine learning solutions reflect the strength of a prediction even for binary classifications. This graph relates the prediction strength to the performance. The x-axes give the prediction strength as the RI (from -100: very reliable noninteraction to 100: very reliable interaction). The y-axes reflect the precision percentage (red line, Eq 3) and recall percentage (blue line, Eq 2). The precision is proportional to the prediction strengths, i.e., predictions with a higher RI are, on average, better than predictions with a lower RI. For example, for all the gene relationship predictions with RI>80 (black dashed line), approximately 96% are correct predictions. (B) This graph relates prediction strength to performance for negative predictions (noninteractions). For example, for all the negative gene relationship predictions with RI<-80 (black dashed line), approximately 92% are correct predictions. (C) The MCC (Eq 3) was determined for a comparison among different methods on the test set, and our method NECARE obtains the highest MCC: 0.84. (D) ROC curve comparison for different methods based on the test set. NECARE has the largest AUC: 0.97.

Finally, we evaluated the performance of NECARE in the testing set. Overall, NECARE achieved an F1 = 91±2% and an MCC = 0.84±0.03 (S1 Table). In addition, we also determined the reliability index (RI) of NECARE (Fig 3A). RI was correlated with its performance and can be used to measure its prediction performance. The RI ranged from -100 to 100 (-100 meant most reliable negative prediction and 100 meant most reliable positive prediction). For instance, the subset of predictions at RI ≥ 0 had a precision of >90% (Fig 3A: red line at x = 0). This level covered approximately 92% of all predictions (Fig 3A: blue line at x = 0). When increasing the RI to 80 (dashed line), the precision reached 95% (Fig 3A: red line at x = 80), but it can cover only 74% of all predictions (Fig 3A: blue line at x = 80). Therefore, basically, a higher RI represented a more reliable prediction. The RI was also calculated for the negative prediction (noninteracting prediction) (Fig 3B). At RI = 0, the precision for the negative prediction was 94%, and it increased to 97% at RI = -80 (Fig 3B).

### NECARE outperformed other algorithms

As NECARE is a network-based method, we first compared it with other network-based node relationship prediction algorithms such as the state of art method L3 [22], and the methods they compared in their research. We also compared NECARE with other state-of-the-art sequence-based deep learning PPI prediction methods such as PIPR [20] and DPPI [18] (Fig 3C and 3D).

Firstly, we conducted the comparison on training data, drew the ROC (receiver operating characteristic) curves for all the methods (Fig 3D) and calculated the AUC for them. Our method achieved the best performance with an AUC = 0.97 (Fig 3D and S1 Table), while most of the other methods had an AUC of 0.60 approximately (S1 Table). For the detailed metrics, NECARE reached the highest F1 (91±2%) and MCC (0.84±0.03) in the comparison (Fig 3C and S1 Table). The RCNN (recurrent convolutional neural network)-based method PIPR achieved the highest precision of 94±1% (precision for NECARE was 90±2%). However, PIPR had a low specificity of 83±8% and MCC of 0.65±0.10, and the specificity of NECARE was 92±2% (S1 Table).

Secondly, we repeated the comparison on the independent set. Consistent with the results on training set, NECARE achieved highest performance in independent comparison with a highest AUC = 0.93±0.20 (S3 Fig).

Overall, we can conclude that NECARE is currently the best prediction method that can be used to identify PPIs in cancer.

### Cancer hub genes discovered by NECARE

By applying NECARE, unlike previous studies that were limited to the coexpression between genes [2], we were able to reveal the comprehensive and rigorous perturbation of the cancer gene network (Fig 2). We mapped the cancer gene interactome with its highly reliable predictions (|RI| ≥ 0.8, Fig 3A and 3B). On average, each gene lost 31 edges in the cancer network. However, they obtained approximately 124 new edges on average (S4 Fig, red dashed lines). This verified our hypothesis that instead of simply being fractured, the network in cancer is reprogrammed.

**Fig 4:**
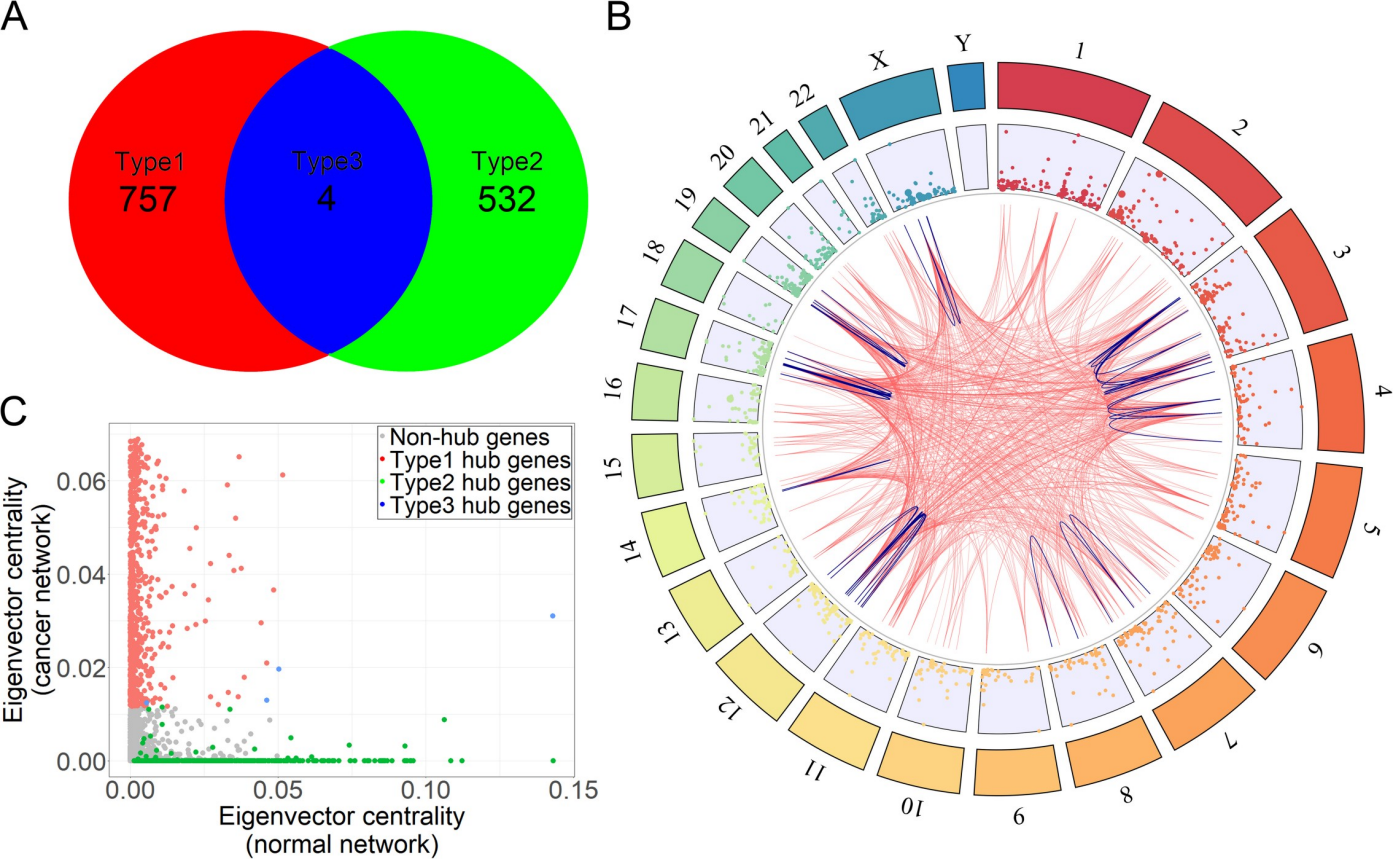
Cancer hub genes of the cancer gene relationship network. Type 1: hub genes enriched for only gained links; Type 2: hub genes enriched for only lost links; Type 3: hub genes enriched for both gained and lost links. (A) The number of three different types of cancer hub genes. (B) The distribution of cancer hub genes among chromosomes. The links inside the circle are the top 1000 links between cancer hub genes based on the NECARE output scores. The blue links were inside-chromosome interactions. (C) The centrality eigenvector of cancer hub genes. The x-axis is the centrality in the normal network, and the y-axis is the centrality in the cancer network.

Furthermore, we assumed that the perturbation was not evenly distributed among all the genes. Some genes may hold more perturbations than others. Genes enriched with network perturbations (gained/lost links) were defined as cancer hub genes. Finally, we identified 1293 genes enriched with network perturbations in cancer (Figs 2 and 4A and S2 Table).

Then, we classified cancer hub genes into three types: Type 1, hub genes enriched with gained links; Type 2, hub genes enriched with lost links; and Type 3, hub genes enriched with both gained and lost links. Overall, we identified 757 Type 1 hub genes, 532 Type 2 hub genes and 4 Type 3 hub genes (Fig 4A). With an interest in the distribution of the PPIs of cancer hub genes in human chromosomes, we showed the distribution of the top 1000 links with highest RI among all the hub genes in chromosomes in Fig 4B. We can see that, in consistent with the uneven chromosome distribution of cancer genes in previous study, the PPIs of cancer hub genes also distributed unevenly [28].

And even among hub genes, the top 1000 links were not distributed evenly, and some hub genes had more links than others. For example, CDK4 was engaged in 150 links among the top 1000 links and EGF was engaged in 109 links. In contrast, 39 hub genes engaged in only one link among the top 1000 links.

Type1 and Type2 hub genes were found enriched in very different pathways. Type 1 hub genes which tend to get new PPIs in cancer network were enriched in a lot of famous oncogenic signaling pathways [29], including: MAPK signaling pathway (P-value = 1.11x10^-27^), PI3K-Akt signaling pathway (P-value = 2.27x10^-17^) and Wnt signaling pathway (P-value = 2.43x10^-18^) (S5A Fig). Many famous cancer genes were Type1 hub genes including BRCA1, CDK1, CDK4, CDK14, EGF, JUN, KRAS, MYC, and YAP1. Meanwhile, Type 2 hub genes which tend to lose PPIs in cancer network were enriched in pathways for more general functions, such as Ribosome biogenesis in eukaryotes and Splicesome. One of the well-known Type 2 hub gene was TP53 (113 interactions lost, S2 Table), which was correspond to the annotation from KEEG database (Fig 1C). Besides, the most interesting result was that the type 2 hub genes were enriched in COVID-19 pathway (S5B Fig). This could be a kind of explanation of the previous finding that having cancer was an independent risk factor for in-hospital death from COVID-19 [30].

4 genes were Type 3 hub genes which had both gained- and lost-link perturbations (Fig 4A and S2 Table), including POLR2B, S100A2, RPL15 and UBE2K. S100A2, which involves a number of cellular processes such as cell cycle progression and differentiation, was dysregulated in lung, gastric, esophageal, ovarian, bladder, breast, thyroid, melanoma and pancreatic cancer [31]. RPL15 was related to the prognosis of different cancers: glioma, breast cancers, gastric cancer, leukemia and pancreatic ductal adenocarcinoma [32–35].

**Fig 5:**
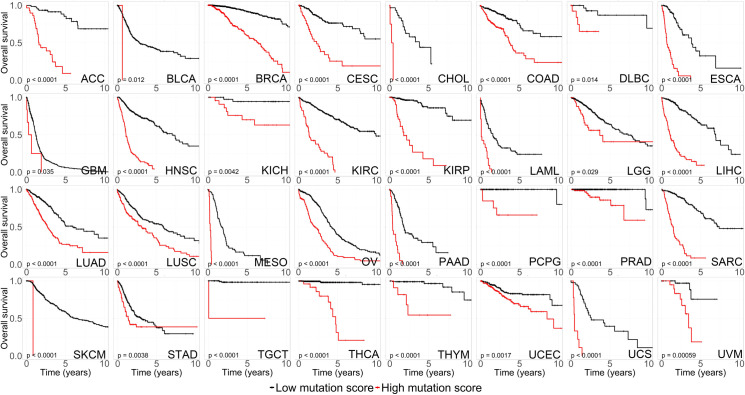
The prognostic landscape of hub genes. Kaplan–Meier plots for the patients from 32 different types of cancers from TCGA divided into high- and low-MS groups (Materials and Methods). The P-value was calculated by the log-rank test.

More interestingly, over 41% of genes that were found to be involved in cancer treatment were cancer hub genes in our study. Among them, 38% were Type 1 hub genes, 3% were Type 2 hub genes. In addition, the distribution of the lost edges had no difference between clinically related genes and the background (all genes) (Kolmogorov–Smirnov P-value = 0.35, S4B Fig). However, there was a significant difference in the distribution of the gained edges (Kolmogorov–Smirnov P-value < 8.5×10^−10^, Mean_All genes_ = 125 and Mean_Clinically related genes_ = 361) (S4A Fig). Furthermore, those hub genes were significantly associated with the 10-year survival outcomes of 32 distinct types of cancer (Fig 5). Overall, patients with high mutation scores had a poor prognosis and low survival rate (red lines in Fig 5).

Subsequently, we analyzed the centrality of those hub genes (Fig 4C). Three types of hub genes and the non-hub genes could be clearly separated by the centrality. This suggested that our statistical analysis, which was applied to identify hub genes, was reliable because we did not consider centrality during the identification of genes. In addition, we found that Type 1 hub genes tended to have a high centrality in the cancer network but a low centrality in the general network. However, Type 2 hub genes showed the reverse trend (a high centrality in the general network but a low centrality in the cancer network). Type 3 hub genes were balanced between Type 1 and Type 2 hub genes. Notably, these non-hub genes had a low centrality in both general and cancer networks. The centrality changes in Type 1 and Type 2 hub genes also reflected the perturbation of the cancer network.

### Experimental validation of NECARE predictions

**Fig 6:**
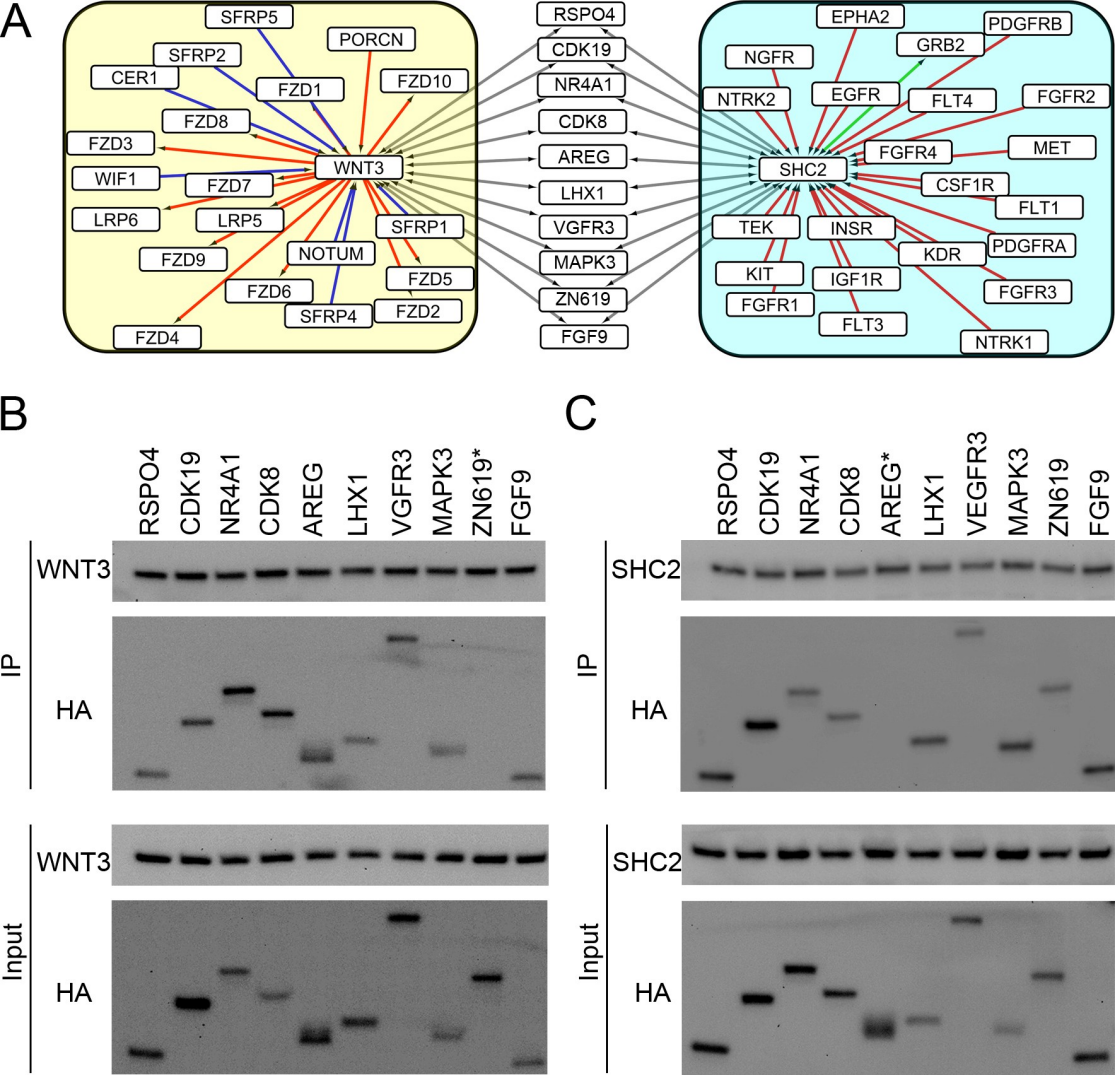
Experimental validation of the NECARE predictions. **Panel A** shows the genes that cross-talk with WNT3 and SHC2 in each pathway. Different colored edges represent different types of interactions. The red edge indicates activation; the blue edge indicates inhibition; the green edge is the KEGG annotated binding; the gray edge is NECARE predicted binding. The left yellow group shows the genes interacting with WNT3 in the Wnt signaling pathway. The right cyan group shows the genes in contact with SHC2 in the Ras signaling pathway. Those 10 genes in the middle with gray edges are NECARE predicted genes binding to WNT3 and SHC2 with a high RI (> 90). **Panels B** and **C** are co-IPs that validated the interactions of 10 predicted genes with WNT3 and SHC2 in LN229 cells. The interactions were determined by immunoblotting. The labelled “*” indicates a negative result of the co-IP validation experiment. **Panel B:** LN229 cells were co-transfected with the indicated HA-tagged constructs of 10 predicted genes and FLAG-tagged WNT3. **Panel C:** LN229 cells were co-transfected with the indicated HA-tagged constructs of 10 predicted genes and FLAG-tagged SHC2.

The Wnt and Ras signaling pathways are two most important pathways in cancer. And there could be a cross-talk between these two pathways. Fig 6A shows 10 highly reliable (RI > 90, Fig 3A) interactions predicted by NECARE between WNT3 (from the Wnt signaling pathway) and SHC2 (from the Ras signaling pathway) with the following genes: RSPO4, CDK19, NR4A1, CDK8, AREG, LHX1, VGFR3, MAPK3, ZN619 and FGF9. WNT3 is a member of the Wnt family and may play a key role in cancer through activation of the Wnt-beta-catenin-TCF signaling pathway [36]. SHC2 was located very upstream of the Ras signaling pathway and could be activated by many receptor tyrosine kinases (RTKs) in the Ras signaling pathway [6] (Fig 6A).

We applied coimmunoprecipitation (co-IP) to validate the predictions (S1 Text, coimmunoprecipitation). We co-transfected the expression vectors of these 10 genes together with WNT3 and SHC2 in glioblastoma cell line LN229 (Fig 6B and 6C). Co-IP was applied to confirm their binding interaction. 90% (18 of 20) of NECARE predictions were confirmed (Fig 6B and 6C). Only two pairs of interactions, ZN619-WNT3 and AREG-SHC2, obtained negative validation results in co-IP (Fig 6B and 6C).

## Discussion

Previous studies have already found that somatic missense mutations were significantly enriched in PPI interfaces compared to non-interfaces and those mutations would have “edgetic” effect to alter the PPIs [37,38]. Meanwhile, some other study confirmed several co-expression network perturbations in cancer [2]. All these results indicated that the PPI network in cancer might be different from that in non-disease situations. In our study, we used R-GCN to establish a PPI prediction method, NECARE, which is specific for cancer.

In the biological cell system, instead of isolation, genes act as a complex network. Genes may be regulated by others, control the expression of many other genes, or function together with other genes. Our model simulated this biological system by using a R-GCN, which uses the gene network information containing directions and types to predict the PPIs in cancer. Then, we compared our method with other two kinds of algorithms: 1) sequence-based methods and 2) network-based methods. Our system outperformed all other algorithms in the task of predicting PPIs in cancer. Sequence-based, state-of-the-art methods, such as PPI-Detect and PIPR [19,20], achieved good performance in PPI prediction of non-disease condition but failed in our cancer-specific task. Since proteins were acting as a network complex, the disorder information would be broadcasted among the network. And the interaction between two proteins may also be affected by their neighbors in the network. Therefore, sequence-based methods which only considered the input proteins themselves may not be very specific for cancer PPI prediction. This is also the reason why we used network-based algorithm combined with knowledge-based features such as OPA2Vec. Our system with R-GCN can use the information of types and directions of gene relationship to predict PPIs in cancer, while other network-based algorithms are not able to do so. Thus, our method is currently the best solution for cancer PPIs prediction.

With the help of NECARE, we identified 1293 cancer hub genes that were enriched with network perturbations in cancer. As gene network perturbation was already found to be the main reason for cancer, these cancer hub genes should be the focus of the pathological mechanisms and treatment targets. Indeed, we found that a high mutation score of hub genes was significantly related to a poor prognosis of 32 different types of cancers. Almost half of the cancer treatment-related genes in the database TARGET were hub genes in our study. Thus, these hub genes we identified have a high potential to be the drug design targets for cancer treatment and the other clinical research.

In addition, as mentioned before, we classified the hub genes into three types: Type 1 (gained links), Type 2 (lost links), and Type 3 (both gained and lost links). Unexpectedly, a lot of famous cancer genes were Type1 hub genes, and previous clinical studies also focused more on these hub genes. This phenomenon may be corresponding to the fact that cancer cells have their special characteristics, like limitless replicative potential, sustained angiogenesis and tissue invasion and metastasis. Gained links of genes in the network will lead to the new functions of the whole cellular system, which can in some extent explain the behavioral characters of cancer cells. This can also explain why previous clinical studies also focused more on these hub genes. Targeting the newly established PPI in cancer cells may inhibit the new functions obtained by them, which can further block the uncontrolled proliferation, migration and invasiveness of cancer cells. Actually, there are also some famous cancer related genes, which not only get a lot of new interactions but also lose some links with other genes in cancer network. These results are corresponding to the previous studies that, instead of the simple destruction, cancer mutations lead the reconstruction of the PPI network and those mutations located in PPI interfaces are highly correlated with patient survival [7,37]. So, as a new perspective of cancer research which may lead to a better understanding of the pathological mechanism of cancer, we should also focus on how the cancer genes reprogram the PPI network with both the links they lose and the new interaction they get. Maybe this will provide a treatment strategy for those intractable cancers.

Overall, in our study, we established the first cancer-specific PPI prediction method. With the help of our new method, we analyzed PPI network perturbations in cancer and identified cancer hub genes. Our method provides a powerful tool for biology researchers and clinicians to find possible interacting partners of their input proteins in cancer. They can also choose to focus their research on the cancer hub genes identified by our method to develop new targets for cancer treatment.

## Materials and methods

### General gene relationship data

To predict cancer PPIs with R-GCN, we need to build a knowledge graph which contained information of the relationship between genes (Fig 2). In order to build the knowledge graph, we extracted the general gene network data from the following three databases:1) STRING [39], a famous database for known protein-protein associations, from which we extracted data about the experimental annotated human protein-protein associations; 2) Kyoto Encyclopedia of Genes and Genomes (KEGG) [6], a well-known publicly accessible pathway database, from which we extracted human non-disease pathway; and 3) HIPPIE [40], which contains experimentally detected PPIs from IntAct [41], MINT [42], BioGRID [43], HPRD [44], DIP [45], BIND [46] and MIPS [47]. Overall, our general gene relationship data contained 551850 pairs of interactions (S3 Table). The whole dataset is available from (github.com/JiajunQiu/NECARE/dataset/NECARE.graph).

### Cancer protein-protein interaction data

Cancer protein-protein interaction data served as the training data for the R-GCN (Fig 2). We obtained cancer PPI data from the KEGG and Reactome databases [6,48], which served as the positive training set. We also included the OncoPPI database [7], which is an experiment-based cancer-specific PPI database, in our positive training set. The negative training data were the pairs of relationships with “disassociation/missing interaction” or other negative annotations in the KEGG cancer related pathways.

Overall, we have 933 positive interactions (links) and 1308 negative interactions (links). The whole dataset is available from (github.com/JiajunQiu/NECARE/dataset/NECARE_TrainingData.txt).

### The 5-fold cross-validation

We applied a 5-fold cross-validation approach for the training process (Figs 2 and S2). Technically, we divided the training set into five parts. In each rotation, we used three of the five parts for training, one for cross-training (optimize hyperparameters, including number of hidden units in neural network, early stop, etc.), and one for testing. Overall, we train the models with different hyperparameters and features on training set, and we picked the combination with best performance on the cross-training set (S4 Table). Finally, we evaluated the final performance on the testing set. The testing set was never used in the hyperparameter optimization and feature selection.

### Relational graph convolutional networks

Graph convolutional networks (GCNs) can be understood as special cases of a simply differentiable message-passing framework. Information can be obtained from the neighbors of each node in the GCN. The R-GCN is an extension of the GCN [49]. It accounts for the edge type and direction and can calculate the forward-pass update of an entity or node denoted in relational (directed and labeled) multigraphs [49] (Fig 1D).


$$h_{i}^{\left(l+1\right)}=\sigma \left(\sum_{r\in R}\sum_{j\epsilon N_{i}^{r}}\frac{1}{C_{i,r}}W_{r}^{\left(l\right)}h_{j}^{\left(l\right)}+W_{O}^{\left(l\right)}h_{i}^{\left(l\right)}\right)$$ (1)

In Eq 1, if we define the directed and labeled multigraphs as G=(V,E,R) with the nodes defined as $v_{i}\;\epsilon V$, labeled edges as (vi,r,vj)ϵE, and edge type as rϵR, then $h_{i}^{\left(l\right)}$ is the hidden state of node v_i_ in the i-th layer of the neural network. $N_{i}^{r}$ denotes the set of neighbor indices of node v_i_ under the relation rϵR. *C_i,r_* is a normalization constant, which is defined as the degree of the target node of an edge. $W_{r}^{\left(l\right)}$ is a form of weight sharing among different relation types, and $W_{O}^{\left(l\right)}$ is a weight matrix for the linear message transformation. The incoming messages from neighbors are accumulated and then passed through an activation function σ such as ReLU [49]. Therefore, in our study, instead of only considering the gene itself, information about each gene was obtained from other genes that contacted it.

Regarding to the feature we used to train the model, it was a combination of two parts. Part one was the OPA2Vec vector of each gene, which was a knowledge-based feature [50]. OPA2Vec is a tool that can be used to produce feature vectors for biological entities from ontology. OPA2Vec used mainly metadata from the ontology in the form of annotation properties as the main source of data. In this study, we used the OPA2Vec pretrained model based on PubMed data, and the annotation file was downloaded from http://purl.obolibrary.org/obo/go.owl. Part two was the cancer-specific feature based on The Cancer Genome Atlas (TCGA), including the expression profile of each gene in 32 different types of cancer and the mutation rate among patients for each type of cancer.

### Performance evaluation

We evaluated the performance of the prediction via a variety of measures. For simplicity, we used the following standard annotations: true positives (TP) were the correctly predicted gene relationships in cancer, while false positives (FP) were the gene pairs that had no links in cancer and were incorrectly predicted to have interactions. True negatives (TN) were the correctly predicted noninteractions, and false negatives (FN) were the gene pairs that had interactions but were not correctly predicted.


$$\begin{array}{r} \mathrm{Precision}=\mathrm{TP}/(\mathrm{TP}+\mathrm{FP});\;\mathrm{Sensitivity}\;(\mathrm{Recall})=\mathrm{TP}/(\mathrm{TP}+\mathrm{FN})\\ \mathrm{Specificity}=\mathrm{TN}/(\mathrm{TN}+\mathrm{FN}) \end{array}$$ (2)

$$F1=2^{*}{\mathrm{Precision}}^{*}\mathrm{Recall}/(\mathrm{Precision}+\mathrm{Recall})$$

We also calculated the Matthews correlation coefficient (MCC) and area under the curve (AUC):

$$MCC=\frac{TP\times TN-FP\times FN}{\sqrt{\left(TP+FP)(TP+FN)(TN+FP)(TN+FN\right)}}$$ (3)

### Error estimates

Error rates for the evaluation measures were estimated by bootstrapping (without replacement to render more conservative estimates), i.e., by resampling the set of samples used for the evaluation 1000 times and calculating the standard deviation of those 1000 different results. Each of these sample sets contained 50% of the original samples (picked randomly again, without replacement).

### Comparison with other methods and the independent data set

The comparison with other methods were conducted on both training and independent dataset. The independent dataset was created based on literature-curated experiment results, which contains overall 229 cancer PPI annotations (github.com/JiajunQiu/NECARE/dataset/NECARE_IndependentData.txt).

And we compared two different kinds of PPI prediction methods and fed them with related inputs: 1) sequence-based methods. Sequence-based methods took the sequences of two proteins as input and used the features such as chemical-physical properties of amino acids (Method: PPI-Detect) to predict the interaction between proteins. 2) Network-based methods. Network-based methods took the mapped interaction network as input and exploited the patterns characterizing the network to identify the interaction among the nodes. For example, method L3 predicted the interaction between two nodes by using paths of length 3 which connects two nodes in the input network.

### Cancer hub gene identification

Cancer hub genes were defined as those genes that significantly lost (or gained) links in the cancer network, compared with the general network. Thus, to identify the cancer hub genes, we need two different networks: cancer PPI network and non-disease general network. Cancer PPI network was predicted by NECARE, while the general PPI network was defined by two parts:1) first, we extracted the literature-based general PPI network from the general gene network which was used in the training process of NECARE; 2) Literature curated interactomes of PPIs, which have excellent replicability, but are impacted by selection biases. To solve such problem, according to the previous publication [22], we also consider interactomes emerging from systematic screens, that lack such biases [51–54].

We used the cancer gene links connecting with an equal likelihood at the genes in the network as a null model. We assumed that, for a particular gene (node) to be called a putative hub gene, more links (gained or lost) must connect to that gene than expected by chance if the links were randomly connected to the genes in the network. Randomly, the frequency of links connected to any particular residues followed a binomial distribution:

P(m=k)=(nk)pk(1−p)n−k (4)

where n is the 2x total number of links, k is the number of links connecting to a particular node, p is the probability of any individual link connecting at a particular node, and P (m = k) is precisely the probability of observed k links at a single node. Since our null model assumes an equal likelihood of links at any node, we used p = 2/L, where L is the overall number of unique nodes in the network.

Thus, to assign a probability to the observation of k links connecting at a particular node by chance (i.e., a P-value), we calculated the probability of at least k links connecting at a particular node from our null model:

P(m≥k)=∑i=kn(nk)pk(1−p)n−k (5)

To correct for and test multiple hypotheses, the p-values for all considered hub genes were adjusted using the Bonferroni correction method.

Eigenvector centrality was a measure of the influence of a node in a network. The regular eigenvector centrality of each gene in the network was the eigenvector of the adjacency matrix with the largest unique eigenvalue. Here, in our study, we applied a variant of eigenvector centrality [55]. The final centrality values followed the SoftMax probability: any node that you randomly picked up would reach a certain node in the network.

### Clinically related cancer genes

Cancer genes related to clinical treatment were downloaded from the Tumor Alterations Relevant for GEnomics-driven Therapy (TARGET) database (https://software.broadinstitute.org/cancer/cga/target). TARGET (tumor alterations relevant for genomics-driven therapy) is a database of genes that, when somatically altered in cancer, are directly linked to a clinical action. TARGET genes are associated with response or resistance to a therapy, diagnosis, and/or prognosis.

### Survival analysis of hub genes

To assess the association of hub genes with survival outcomes, we obtained the mutation and clinical prognosis data of 32 different types of cancers from the TCGA (S5 Table). For each cancer, we first calculated hazard ratios (HRs) and P-values (log-rank test) for each involved gene by Cox proportional hazards regression analysis using the coxph function of the R survival package (v. 2.37.2). Then, for each cancer, we integrated the hub genes with a significant P-value (cutoff: 0.05) into a combined mutation score (MS):

$$MS=\sum_{j=1}^{g}\left(w_{j}\times M_{j}\right)$$ (6)

where *M*_*j*_ is whether gene *j* is mutated in the tumor sample of the patient (1 for mutated and 0 for nonmutated) and *W*_*j*_ is set to 1 or -1, depending on the HR of each gene (1 for HR ≥ 1 and -1 for HR<1). The median value (50%) or the automatically selected best cutoff value of the MS was used to divide the corresponding patients into high- and low-MS groups for Kaplan–Meier analysis of their association with the 10-year survival.

## Supporting information

S1 Text Experimental validation of NECARE. (DOC)

S1 Table Summary of the comparison based on test set. (DOC)

S2 Table Cancer hub genes (tsv). (TSV)

S3 Table Summary of general gene relationship data. (DOC)

S4 Table Cancer names in survival analysis from TCGA. (DOC)

S5 Table Optimized hyperparameter of NECARE in cross-training set. (DOC)

S1 Fig Performance comparison among different training processes of NECARE on the cross-training set. **OPA2Vec+TCGA (RGCN):** was the one used in final version of NECARE, which used general gene network in the input end, took the information of the link directions and types into consideration and used the OPA2Vec+TCGA as the input features. OPA2Vec+TCGA (RGCN*): instead of the general gene network, it means training NECARE with only general PPI network, excluding the interactions such as expression regulation. **OPA2Vec+TCGA (GCN):** training NECARE without the information of the link directions and types. **OPA2Vec:** using the ontology-based feature OPA2Vec alone. **TCGA:** means using only the TCGA-based expression and mutation profile.
(TIF)

S2 Fig Cross-validation procedure. For all machine learning developments, the original nonredundant data were split into five parts (Part 1-Part 5). Three parts were used for training, one for cross-training (optimization of hyperparameters, choice of feature), and one for testing. This was repeated five times (Fold 1-Fold 5, 5-fold cross-validation) so that each protein in the original data set had been used exactly once in the training set. Estimates for the standard error were compiled through bootstrap (Materials and Methods), not as the difference between the five folds.
(TIF)

S3 Fig ROC curve comparison for different methods based on the independent set. NECARE has the largest AUC: 0.93.
(TIF)

S4 Fig Distribution of gained or lost edges. (A) The distribution of gained edges. The dashed lines represent the mean. Mean_All genes_ = 125 and Mean_Clinically related genes_ = 361. (B) The distribution of lost edges. The dashed lines represent the mean. Mean_All genes_ = 30 and Mean_Clinically related genes_ = 31.
(TIF)

S5 Fig KEGG enrichment analysis for cancer hub genes. The x-axis is the gene ratio, which represents the percentage of all genes annotated to a pathway. Dot size is the number of genes annotated to a pathway. The color of each dot corresponds to the P-value of KEGG enrichment analysis. (A) KEGG enrichment analysis for Type1 hub genes. (B) KEGG enrichment analysis for Type2 hub genes.
(TIF)
