# Supplementary material for: Network-based protein-protein interaction prediction method maps perturbations of cancer interactome
Source: PLoS Genet. 2021 Nov 2;17(11):e1009869. doi: 10.1371/journal.pgen.1009869 (PMC8610286; doi:10.1371/journal.pgen.1009869)
Supplement: S1 Text — (DOC) [file pgen.1009869.s001.doc]

S1 Text: Experimental validation of NECARE.

**Cell culture and reagents**

The commonly used glioblastoma cell line LN229 (ATCC, USA) was cultured with Dulbecco’s Modified Eagle Medium (DMEM) full medium (DMEM, 10% FBS, 0.1 mg/mL streptomycin, 100 U/mL penicillin, and 0.025 mg/mL amphotericin B) and was maintained in 5% CO_2_ atmosphere at 37°C.

**Plasmid constructs and transfection**

FLAG-WNT3 and FLAG-SHC2 were cloned into the pcDNA5 expression vector. Meanwhile, HA-RSPO4, HA-CDK19, HA-NR4A1, HA-CDK8, HA-AREG, HA-LHX1, HA-VGFR3, HA-MAPK3, HA-ZN619 and HA-FGF9 were subcloned into the pcDNA3.1 vector. LN229 cells were grown to be 70-80% confluent in 6-cm dishes before transfection. Then, they were transfected with Lipofectamine 3000 (Invitrogen) following the manufacturer’s protocol. Cells were typically analyzed 24-36 h posttransfection.

**Western blot analysis**

The protein content of cell lysates was determined by western blot, and approximately 30 μg of whole lysates was used for each sample. In brief, whole lysates were separated by SDS polyacrylamide gel electrophoresis (SDS-PAGE) and transferred onto a polyvinylidene fluoride (PVDF) membrane. After blocking in 5% milk for 30 min, PVDF membranes were incubated overnight at 4°C with the corresponding primary antibodies: anti-FLAG (1:1000, Abcam), anti-HA (1:1000, Abcam) and anti-tubulin (1:2000, Abcam). The primary antibodies were detected with their corresponding horseradish peroxidase-conjugated secondary antibodies. The protein signals were detected using the enhanced chemiluminescence (ECL) substrate (Biocompare).

**Coimmunoprecipitation (co-IP)**

For co-IP, cells were harvested, and whole cell lysates were prepared with lysis buffer (50 mM Tris HCl, pH 7.4, 150 mM NaCl, 1 mM EDTA, and 1% Triton X-100). Then, whole cell lysates were sonicated and centrifuged for 10 min at 4°C at 11000 rpm. Flag-tagged proteins were purified anti-FLAG M2 agarose affinity gel (Sigma-Aldrich). After 2 h of incubation at 4°C, the FLAG immunoprecipitates were washed four times in lysis buffer and eluted with 1 × SDS sample buffer by boiling at 95°C for 10 min. Precipitated proteins were analyzed by western blotting.
