# Supplementary material for: Network-based protein-protein interaction prediction method maps perturbations of cancer interactome
Source: PLoS Genet. 2021 Nov 2;17(11):e1009869. doi: 10.1371/journal.pgen.1009869 (PMC8610286; doi:10.1371/journal.pgen.1009869)
Supplement: S1 Table — (DOC) [file pgen.1009869.s002.doc]

S1 Table: Summary of the comparison based on test set

| Method | AUC | Precision  (%) | Recall  (Sensitivity,%) | Specificity  (%) | F1 (%) | MCC |
| --- | --- | --- | --- | --- | --- | --- |
| NECARE | 0.97±0.02 | 90±2 | 92±2 | 92±2 | 90±2 | 0.84±0.03 |
| AA | 0.71±0.02 | 51±1 | 94±2 | 35±1 | 66±1 | 0.33±0.03 |
| CAA | 0.67±0.02 | 54±2 | 77±2 | 54±2 | 64±2 | 0.31±0.03 |
| CAR | 0.67±0.02 | 56±2 | 75±2 | 58±2 | 64±2 | 0.33±0.03 |
| CN | 0.72±0.02 | 51±1 | 93±2 | 36±1 | 66±1 | 0.33±0.03 |
| CPA | 0.52±0.01 | 42±1 | 99±1 | 2±1 | 59±1 | 0.03±0.03 |
| CRA | 0.67±0.02 | 55±2 | 78±2 | 54±2 | 65±2 | 0.33±0.03 |
| Dice index | 0.68±0.02 | 51±1 | 94±2 | 36±1 | 66±2 | 0.34±0.03 |
| DLPPI | 0.50 | 42±1 | 100 | 0 | 59±1 | 0.00 |
| DPPI | 0.60±0.02 | 50±2 | 68±2 | 47±2 | 58±2 | 0.15±0.04 |
| Jaccard index | 0.67±0.02 | 51±1 | 94±2 | 36±1 | 66±1 | 0.34±0.03 |
| L3 | 0.63±0.02 | 44±1 | 99±1 | 9±1 | 61±1 | 0.19±0.03 |
| MCE | 0.48±0.02 | 40±1 | 88±1 | 5±1 | 55±1 | -0.12±0.03 |
| PIPR | 0.94±0.06 | 90±5 | 84±5 | 83±8 | 86±5 | 0.65±0.10 |
| PPI-Detect | 0.56±0.02 | 45±2 | 66±2 | 42±2 | 54±2 | 0.09±0.03 |
| Preferential Attachment | 0.514±0.005 | 42±1 | 100 | 0 | 59±1 | 0.00±0.02 |
| RA | 0.69±0.02 | 52±1 | 89±1 | 40±2 | 65±1 | 0.32±0.03 |

*Note*: ± gives the error estimated by bootstrapping (Methods). Percentages for precision, recall, specificity, and F1 , MCC score (Eqn. 3 and Eqn. 4)
