# Supplementary material for: Network-based protein-protein interaction prediction method maps perturbations of cancer interactome
Source: PLoS Genet. 2021 Nov 2;17(11):e1009869. doi: 10.1371/journal.pgen.1009869 (PMC8610286; doi:10.1371/journal.pgen.1009869)
Supplement: S3 Table — (DOC) [file pgen.1009869.s004.doc]

S3Table: Summary of general gene relationship data

| Relationship Type | Count |
| --- | --- |
| dephosphorylation | 5 |
| ubiquitination | 38 |
| repression | 48 |
| phosphorylation | 90 |
| state change | 110 |
| expression | 3196 |
| post transcriptional modification | 9971 |
| inhibition | 14797 |
| compound | 25070 |
| activation | 41232 |
| catalysis | 46337 |
| reaction | 91341 |
| binding | 319615* |
| Total | 551850 |

*For the genes which bind itself, we only count it once
