# Supplementary material for: Network-based protein-protein interaction prediction method maps perturbations of cancer interactome
Source: PLoS Genet. 2021 Nov 2;17(11):e1009869. doi: 10.1371/journal.pgen.1009869 (PMC8610286; doi:10.1371/journal.pgen.1009869)
Supplement: S5 Table — (DOC) [file pgen.1009869.s006.doc]

S5 Table: Optimized hyperparameter of NECARE in cross-training set

| Name of parameters | Values |
| --- | --- |
| Learning rate^1^ | 0.01 |
| Number of hidden layers | 2 |
| Number of hidden nodes | 200 |
| Number of bases^2^ | 10 |
| Dropout | 0.2 |

^1^The learning rate for first 30 epoch was set as 0.1, and it was slow down to 0.01 after 30 epoch.

^2^ Basis decomposition was applied to reduce model parameter size and prevent overfitting in RGCN(1). Number of bases would be a common divisor for the number of hidden nodes and the length of features. To make the optimization easier, we shrank the length of final features to be 200 through PCA.
