# Supplementary figures and images for: Network-based protein-protein interaction prediction method maps perturbations of cancer interactome
Source: PLoS Genet. 2021 Nov 2;17(11):e1009869. doi: 10.1371/journal.pgen.1009869 (PMC8610286; doi:10.1371/journal.pgen.1009869)

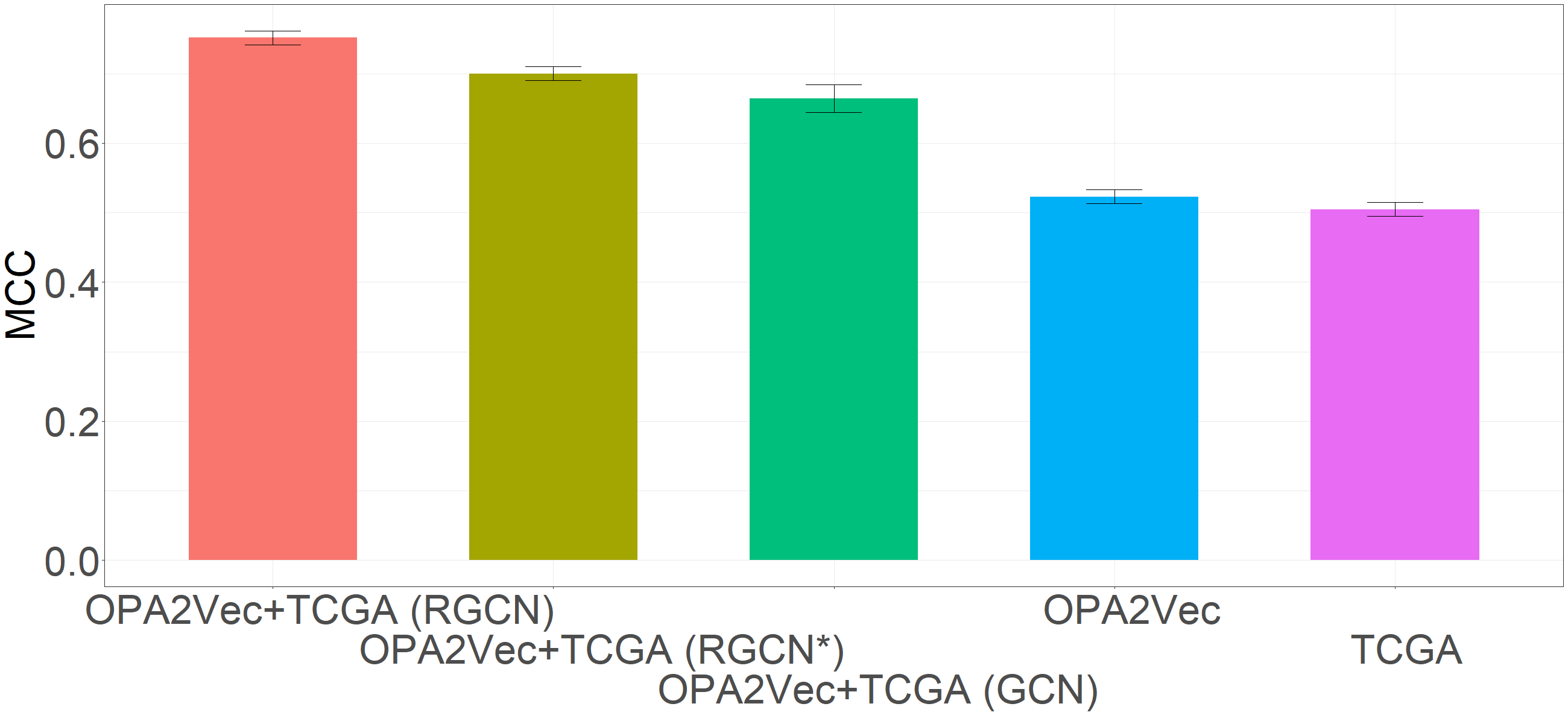

Supplement: S1 Fig — OPA2Vec+TCGA (RGCN): was the one used in final version of NECARE, which used general gene network in the input end, took the information of the link directions and types into consideration and used the OPA2Vec+TCGA as the input features. OPA2Vec+TCGA (RGCN*): instead of the general gene network, it means training NECARE with only general PPI network, excluding the interactions such as expression regulation. OPA2Vec+TCGA (GCN): training NECARE without the information of the link directions and types. OPA2Vec: using the ontology-based feature OPA2Vec alone. TCGA: means using only the TCGA-based expression and mutation profile. (TIF) [file pgen.1009869.s007.tif]

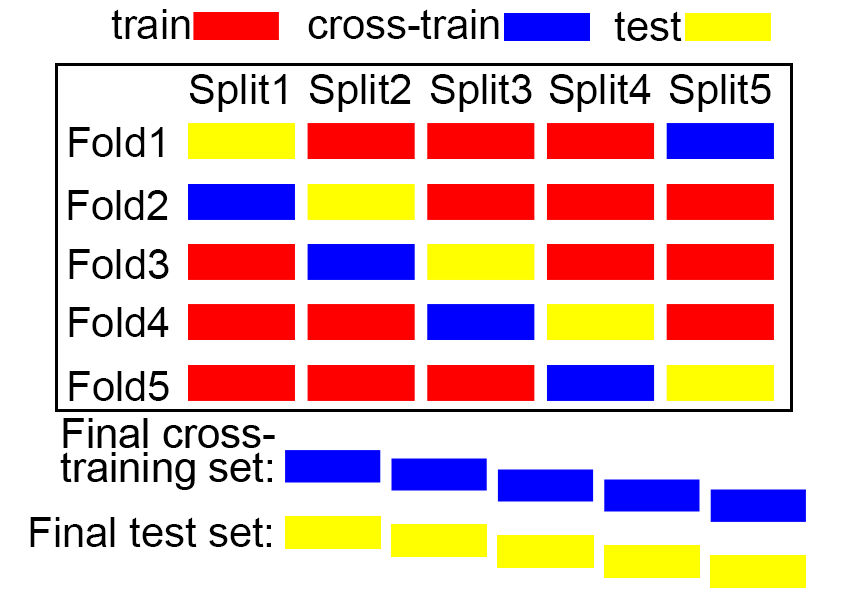

Supplement: S2 Fig — For all machine learning developments, the original nonredundant data were split into five parts (Part 1-Part 5). Three parts were used for training, one for cross-training (optimization of hyperparameters, choice of feature), and one for testing. This was repeated five times (Fold 1-Fold 5, 5-fold cross-validation) so that each protein in the original data set had been used exactly once in the training set. Estimates for the standard error were compiled through bootstrap (Materials and Methods), not as the difference between the five folds. (TIF) [file pgen.1009869.s008.tif]

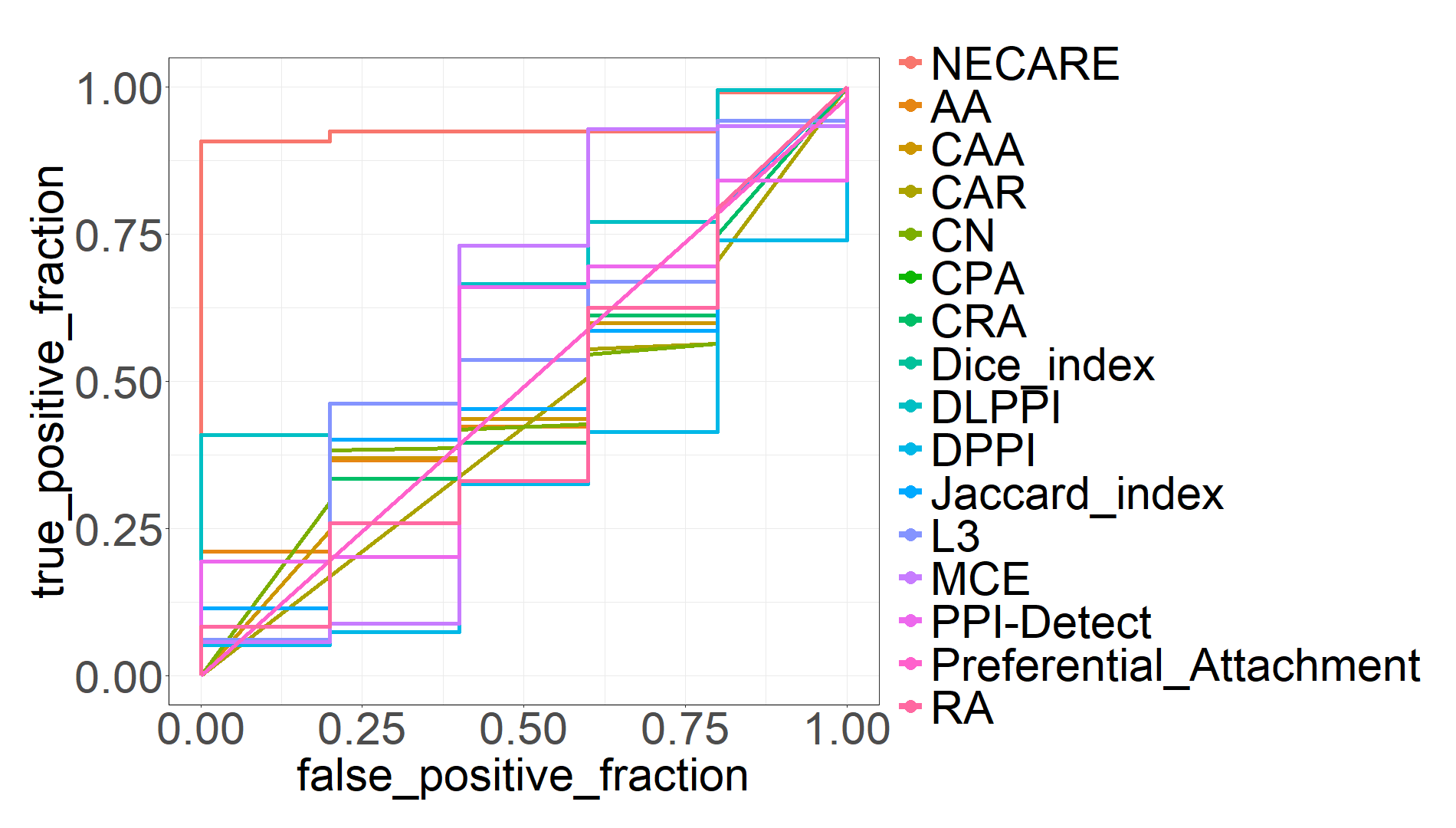

Supplement: S3 Fig — NECARE has the largest AUC: 0.93. (TIF) [file pgen.1009869.s009.tif]

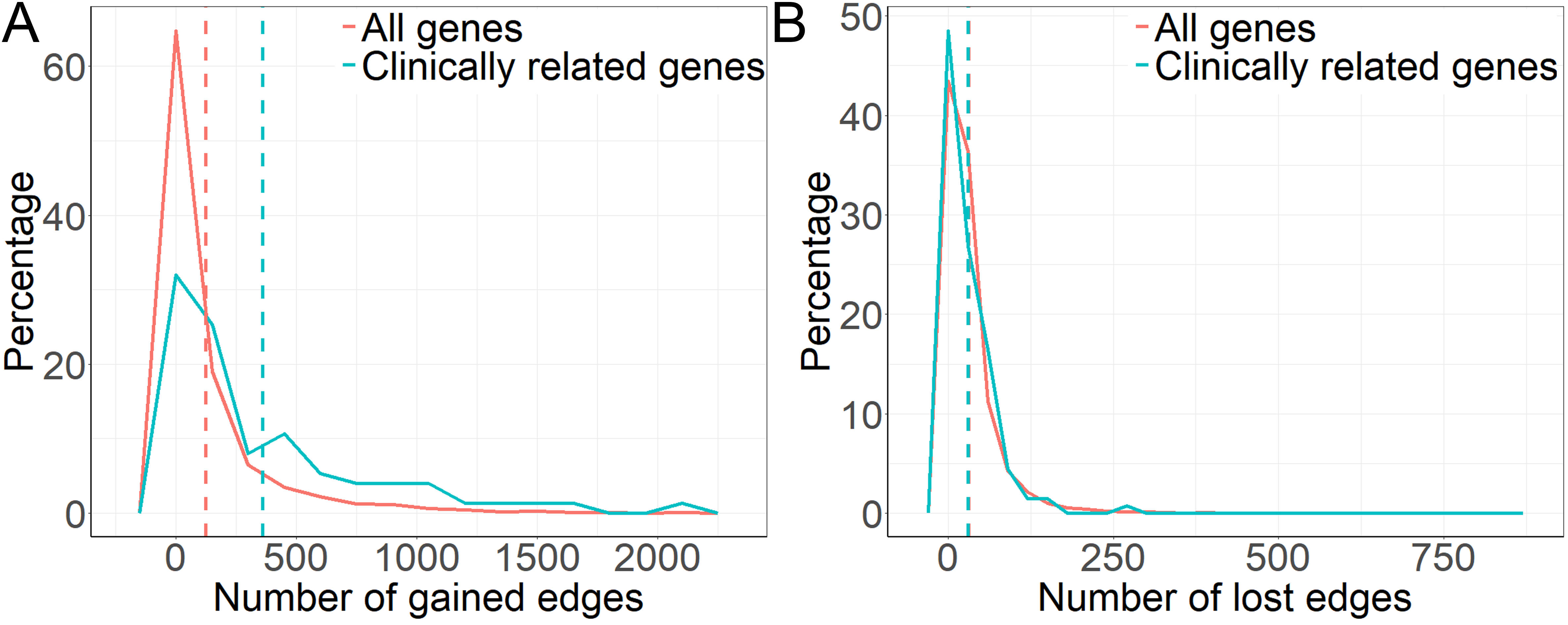

Supplement: S4 Fig — (A) The distribution of gained edges. The dashed lines represent the mean. MeanAll genes = 125 and MeanClinically related genes = 361. (B) The distribution of lost edges. The dashed lines represent the mean. MeanAll genes = 30 and MeanClinically related genes = 31. (TIF) [file pgen.1009869.s010.tif]

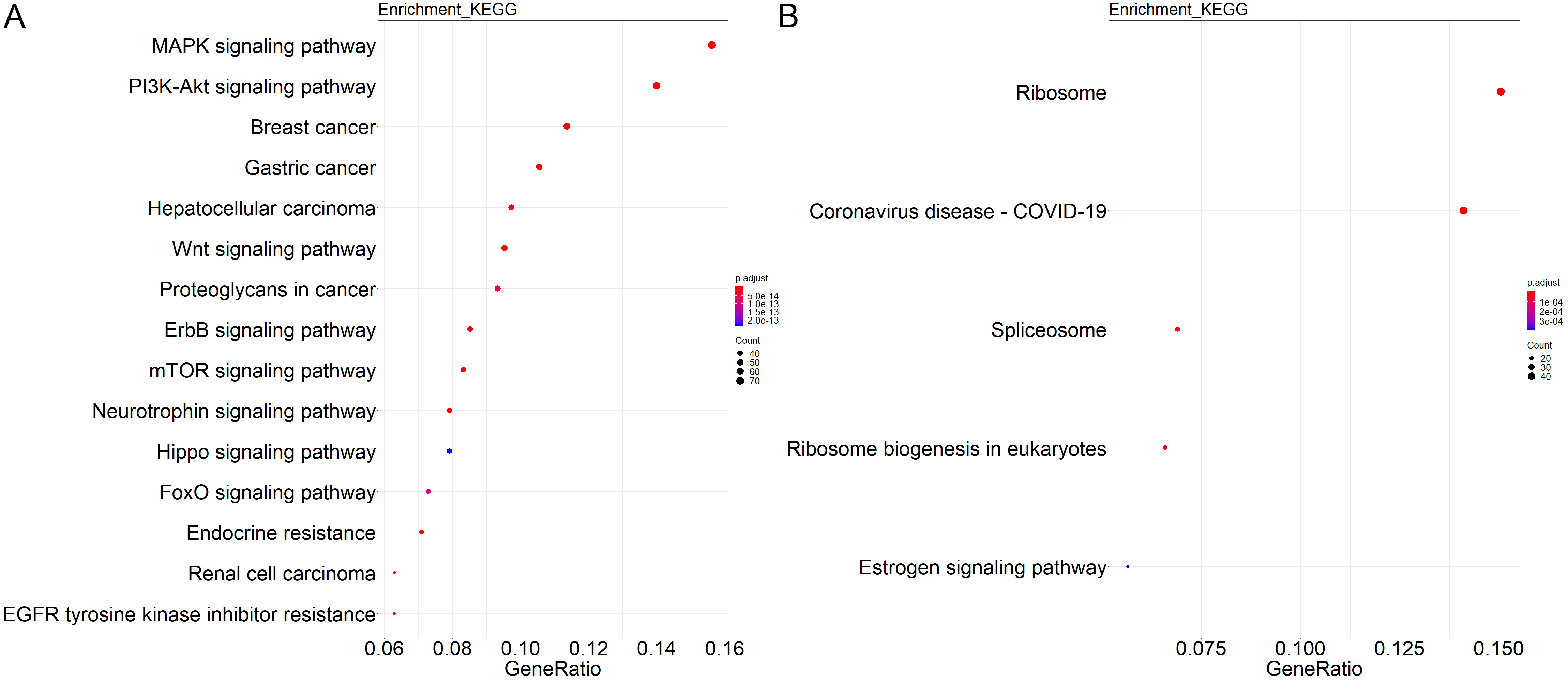

Supplement: S5 Fig — The x-axis is the gene ratio, which represents the percentage of all genes annotated to a pathway. Dot size is the number of genes annotated to a pathway. The color of each dot corresponds to the P-value of KEGG enrichment analysis. (A) KEGG enrichment analysis for Type1 hub genes. (B) KEGG enrichment analysis for Type2 hub genes. (TIF) [file pgen.1009869.s011.tif]
